# Steroidal Saponins Isolated from the Rhizome of *Dioscorea tokoro* Inhibit Cell Growth and Autophagy in Hepatocellular Carcinoma Cells

**DOI:** 10.3390/life11080749

**Published:** 2021-07-26

**Authors:** Shinya Okubo, Tomoe Ohta, Yukihiro Shoyama, Takuhiro Uto

**Affiliations:** Graduate School of Pharmaceutical Sciences, Nagasaki International University, 2825-7 Huis Ten Bosch-Cho, Sasebo, Nagasaki 859-3298, Japan; okubo@niu.ac.jp (S.O.); ohta@niu.ac.jp (T.O.); shoyama@niu.ac.jp (Y.S.)

**Keywords:** autophagy, hepatocellular carcinoma, HepG2, LC3-II, Beclin 1, p62, *Dioscorea tokoro*, steroidal saponins, dioscin, Kampo medicines

## Abstract

Our preliminary screening identified an extract from the rhizome of *Dioscorea tokoro*, which strongly suppressed the proliferation of HepG2 hepatocellular carcinoma cells and inhibited autophagy. This study aimed to isolate active compounds from the rhizome of *D. tokoro* that exert antiproliferative effects and inhibit autophagy. The bioassay-guided fractionation of the active fraction led to the isolation of two spirostan-type steroidal saponins, dioscin (**1**) and yamogenin 3-*O*-*α*-l-rhamnopyranosyl (1→4)-*O*-*α*-l-rhamnopyranosyl(1→2)-*β*-d-glucopyranoside (**2**), and the frostane-type steroidal saponin protodioscin (**3**) from the *n*-BuOH fraction. Furthermore, acid hydrolysis of **1** and **2** produced the aglycones diosgenin (**4**) and yamogenin (**5**), respectively. Compounds **1**–**5** suppressed proliferation of HepG2 cells. The analysis of structure-activity relationships indicated that the 25(*R*)-conformation, structures with a sugar moiety, and the spirostan-type aglycone moiety contributed to antiproliferative activity. Analysis of autophagy-related proteins demonstrated that **1**–**3** clearly increased the levels of both LC3-II and p62, implying that **1**–**3** deregulate the autophagic pathway by blocking autophagic flux, which results in p62 and LC3-II accumulation. In contrast, **1**–**3** did not significantly affect caspase-3 activation and PARP cleavage, suggesting that the antiproliferative activity of **1**–**3** occurred independently of caspase-3-mediated apoptosis. In summary, our study showed that **1**–**3**, active compounds in the rhizome of *D. tokoro*, suppressed cell proliferation and autophagy, and might be potential agents for autophagy research and cancer chemoprevention.

## 1. Introduction

Autophagy is an evolutionarily conserved intracellular process and is considered to be one of the main catabolic pathways [1,2,3,4]. The autophagy machinery consists of several sequential steps: initiation, nucleation of the autophagosome, autophagosome formation and maturation, closure and fusion with the lysosome, and proteolytic degradation of the internalized material [1,2,3,4]. The microtubule-associated protein 1 light chain 3 (LC3) and sequestosome 1 (SQSTM1/p62, herein designated as p62) are associated with autophagosomal membranes that engulf cytoplasmic content for subsequent degradation. LC3, which forms a particularly stable bond with the phagophore, is widely used as a standard marker for autophagy [5,6,7]. The precursor of LC3 is immediately cleaved by cysteine proteases to form LC3-I [5,7]. Phosphatidylethanolamine covalently binds to LC3-I to form LC3-II, which localizes to phagophore, autophagosome, and autolysosome membranes. p62 directly interacts with LC3 on the isolated membrane, and is subsequently incorporated into autophagosomes and degraded [5,8,9]. Previously, it was reported that p62 was rapidly degraded and its levels were decreased when autophagy was induced by starvation conditions. The levels of p62 were restored to basal levels after several hours in HepG2 hepatocellular carcinoma cells [10]. Conversely, p62 accumulates when autophagy is inhibited [11]. Therefore, fluctuations in p62 levels are widely used as indicators of autophagic flux, including the stage of autophagy-mediated degradation.

Several studies have demonstrated the importance of autophagy in various human diseases [2,3,4]. Autophagy occurs at a basal level in all cells and is triggered by various stimuli and cellular stresses. In contrast to normal cells within tissues, tumors are often located in nutrient-, growth factor-, and oxygen-deprived environments as a result of incomplete angiogenesis and uncontrolled growth. In response, tumor cells activate their own autophagic processes to produce amino acids for energy production [12,13,14,15]. Therefore, autophagy is important in supporting tumor growth. In cancers, both the upregulation and downregulation of autophagy are observed, indicating the dual processes of carcinogenesis and tumor suppression during malignant transformation [14,15]. Induction of autophagy may limit tumor development. However, inhibition of autophagy has been observed in mouse models, leading to an enhanced response to anticancer agents through the disruption of tumor defense mechanisms [15,16,17]. Therefore, further studies are needed to understand the modulation of autophagy and cancer development.

Hepatocellular carcinoma (HCC) is the second most common cause of death and the sixth most commonly diagnosed cancer worldwide [18]. In advanced HCC, it has been reported that patients with high LC3 expression have a lower 5-year overall survival rate than patients with low LC3 expression. LC3 is also positively correlated with malignant progression and poor prognosis [19]. Therefore, based on our advanced understanding of the metabolic mechanisms of HCC, therapeutic strategies focused on the modulation of autophagy are expected to contribute to disease eradication.

We previously screened approximately 130 crude drugs used in Kampo formulas in a comprehensive search to identify bioactive compounds that regulate autophagy in hepatocellular carcinoma HepG2 cells [20]. We demonstrated that arctigenin, which is a lignan found in the fruits of *Arctium lappa* and the fruits of *Forsythia suspensa*, inhibited cell proliferation and attenuated autophagy in HepG2 cells [21]. Furthermore, we recently reported the inhibitory effects of costunolide and dehydrocostuslactone, which are sesquiterpene lactones in the root of *Saussurea lappa*, on autophagy [22].

Interestingly, among approximately 130 crude drugs that we screened, the MeOH extract of the rhizome of *Dioscorea tokoro* exhibited the strongest antiproliferative effect on HepG2 cells. Furthermore, the extract significantly increased LC3-II expression and p62 accumulation [20]. *D. tokoro*, a perennial plant belonging to the family Dioscoreaceae, is distributed in the sunny mountains and fields of Japan and in the central to southern part of China. In Japan and China, the rhizome of *D. tokoro*, which is thought to improve the circulation of water in the body, has an anti-inflammatory effect, and is mainly used for treating dysuria, joint pain and skin eczema, although it is rarely used [23]. Chemical analyses have shown that the rhizome contains steroidal saponins, such as dioscin and protodioscin [24,25]. Pharmacological studies have provided evidence that the MeOH extract of rhizomes has the potential to treat rheumatoid arthritis [25]. However, data on the pharmacological activities of the rhizomes of *D. tokoro* and its active compounds are very limited.

In this study, we describe the isolation of active compounds from the rhizomes of *D. tokoro* that exert antiproliferative effects and inhibit autophagy. First, we compared the activity of fractions prepared from the MeOH extract of the rhizome. The bioassay-guided fractionation of the active fractions led to the isolation of several active compounds. Finally, we investigated the effects of isolated compounds on cell proliferation to explore the structure–activity relationship and confirmed the inhibition of autophagy.

## 2. Materials and Methods

### 2.1. General Procedures

Specific rotations were measured using a DIP-360 digital polarimeter (JASCO, Easton, PA, USA). Nuclear magnetic resonance (NMR) spectra were recorded on a JEOL ECX 400 FT-NMR spectrometer (JEOL, Tokyo, Japan) at room temperature. Electrospray ionization time-of-flight mass spectrometry (ESI-TOF-MS) experiments were performed using a Waters Xevo G2-XS Q-TOF mass spectrometer (Waters, Milford, MA, USA). Column chromatography was performed on Silica Gel 60 (Nacalai Tesque, Kyoto, Japan, 230–400 mesh) and YMC ODS-A gel (YMC Co. Ltd., Kyoto, Japan, 50 µm). Thin-layer chromatography (TLC) was performed on TLC Silica gel 60F254 (Merck, Damstadt, Germany) and TLC Silica gel 60 RP-18 F254S (Merck, Damstadt, Germany) plates. The spots were visualized by spraying with 10% aq. sulfuric acid followed by heating. High-performance liquid chromatography (HPLC) was performed using a UV-8020 UV-VIS detector (Tosoh Corp., Tokyo, Japan), DP-8020 pump (Tosoh Corp., Tokyo, Japan), and DP-8020 degasser (Tosoh Corp., Tokyo, Japan). An XBridge BEH C18 Column (Waters, Milford, MA, USA, 130 Å, 3.5 µm, 10 mm × 250 mm) was used for preparative purposes.

### 2.2. Extraction and Isolation

The rhizome of *D. tokoro* (2.5 kg) was extracted three times with MeOH under reflux for 12 h. Evaporation of the solvent under reduced pressure provided a MeOH extract (338.77 g). A portion of the MeOH extract (333.77 g) was partitioned into a hexane–water (H_2_O) (1:1, *v*/*v*) mixture to furnish a hexane-soluble fraction (0.45 g) and an aqueous phase. The aqueous phase was further partitioned into an ethyl acetate (EtOAc)–H_2_O (1:1, *v*/*v*) mixture to furnish an EtOAc-soluble fraction (19.23 g) and an aqueous phase. The aqueous phase was further extracted with *n*-butanol (*n*-BuOH) to yield an *n*-BuOH-soluble fraction (138.24 g) and a H_2_O-soluble fraction (124.17 g). A part of the *n*-BuOH-soluble fraction (21.15 g) was subjected to normal-phase silica gel column chromatography (580 g, chloroform–MeOH–H_2_O (20:3:1 → 15:3:1 → 10:3:1 → 7:3:1 → 6:4:1, *v*/*v*/*v*, lower layer) → MeOH) to give 12 fractions (Fr. B1 (143.2 mg), Fr. B2 (185.2 mg), Fr. B3 (437.6 mg), Fr. B4 (191.5 mg), Fr. B5 (266.9 mg), Fr. B6 (537.3 mg), Fr. B7 (971.1 mg), Fr. B8 (5.14 g), Fr. B9 (2.07 g), Fr. B10 (2.67 g), Fr. B11 (6.24 g), Fr. B12 (1.39 g)). Fraction B8 (5.14 g) was subjected to reversed–phase silica gel column chromatography (305 g, MeOH: H_2_O (7:3 → 8:2 → 9:1, *v*/*v*) → MeOH) to give 14 fractions (Fr. B8–1 (111.5 mg), Fr. B8–2 (32.6 mg), Fr. B8–3 (12.3 mg), Fr. B8–4 (17.4 mg), Fr. B8–5 (18.7 mg), Fr. B8–6 (11.5 mg), Fr. B8–7 (152.1 mg), Fr. B8–8 (3.72 g), Fr. B8–9 (632.2 mg), Fr. B8–10 (15.7 mg), Fr. B8–11 (5.6 mg), Fr. B8–12 (5.2 g), Fr. B8–13 (14.9 mg), Fr. B8–14 (48.5 mg)). Fraction B11 (6.24 g) was subjected to reversed–phase silica gel column chromatography (330 g, MeOH: H_2_O (5:5 → 6:4 → 7:3 → 8:2 →, *v*/*v*) → MeOH) to give 14 fractions (Fr. B11–1 (1.50 g), Fr. B11–2 (50.3 mg), Fr. B11–3 (46.9 mg), Fr. B11–4 (54.8 mg), Fr. B11–5 (221.4 mg), Fr. B11–6 (757.6 mg), Fr. B11–7 (1.10 g), Fr. B11–8 (1.51 g), Fr. B11–9 (485.8 mg), Fr. B11–10 (201.7 mg), Fr. B11–11 (203.6 mg), Fr. B11–12 (139.3 mg), Fr. B11–13 (30.5 mg), Fr. B11–14 (44.8 mg)). A part of the Fr. B8–8 (200.0 mg) was purified by HPLC (acetonitrile: H_2_O: acetic acid (88:12:0.3), XBridge BEH C18) to give **1** (63.9 mg, purity >92%) and **2** (34.2 mg, purity >90%). A part of the Fr. B11–8 (1.51 g) was purified by HPLC (acetonitrile: H_2_O: acetic acid (88:12:0.3), XBridge BEH C18) to give **3** (29.4 mg, purity >95%). The known compounds were identified by comparison of their physical data ([*α*]_D_, ^1^H-NMR, ^13^C-NMR, and MS) with reported values.

### 2.3. Acid Hydrolyses

Compounds **1** and **2** (10.1 and 10.4 mg) were dissolved in 5 mL of 3 mol/L sulfuric acid, and each solution was heated at 90 °C for 6 h. After neutralization with sodium hydroxide, 5 mL of ethyl acetate was extracted from each hydrolysate. Evaporation of the solvent under reduced pressure yielded **4** (5.6 mg from **1**, purity >95%) and **5** (6.0 mg from **2**, purity >81%). Each compound was identified by comparison of their physical data ([*α*]_D_, ^1^H-NMR, ^13^C-NMR, and MS) with reported values.

### 2.4. Reagents for Bioassays

Antibodies against LC3B, p62, caspase-3, and poly (ADP-ribose) polymerase (PARP) were obtained from Cell Signaling Technology (Beverly, MA, USA). Antibodies against β-actin and RIPA lysis buffer were purchased from Santa Cruz Biotechnology (Santa Cruz, CA, USA). Fetal bovine serum (FBS) was purchased from Gibco (Grand Island, NY, USA). All other chemicals were obtained from FUJIFILM Wako Pure Chemical Corporation (Osaka, Japan).

### 2.5. Cell Culture and Treatment

HepG2 cells were grown in Dulbecco’s modified Eagle’s medium (DMEM, regular medium) containing 10% FBS and 1% penicillin-streptomycin-l-glutamine and incubated at 37 °C in a 5% CO_2_ fully humidified atmosphere. During cell treatments, the concentration of dimethyl sulfoxide (DMSO) in the cell culture medium did not exceed 0.2% (*v*/*v*), and the controls were treated with the same concentration of DMSO.

### 2.6. Determination of Cell Viability

Cell viability was measured using a previously described method [20,21,22]. Briefly, cells (0.9 × 10^4^ cells/well) were seeded in 96-well plates. After replacing the regular medium with fresh medium, the cells were treated with each sample at the indicated concentrations and time periods. At the end of treatment, 10 μL of 5 mg/mL 3-(4,5-dimethylthiazol-2-yl)-2,5-diphenyltetra-zolium bromide (MTT) solution was added to each well, and the cells were incubated for another 4 h. The precipitated MTT-formazan crystals were dissolved in 100 μL of 0.04 N HCl-isopropanol, and the amount of intensity of the purple color was measured at 595 nm using an iMark microplate reader (Bio-Rad, Tokyo, Japan). Cell viability was expressed as a percentage of the control culture.

### 2.7. Western Blot Analysis

Cells (1 × 10^6^ cells/dish) were plated in 6-cm dishes. After replacing the regular medium with fresh medium, the cells were treated with each sample at the indicated concentrations and time periods. Cells were harvested and lysed in RIPA lysis buffer containing protease and phosphatase inhibitors. The protein concentration was determined using a dye-binding protein assay kit (Bio-Rad, Tokyo, Japan). Equal amounts of protein lysate were subjected to SDS-PAGE. The proteins were electrotransferred to polyvinylidene fluoride membranes and detected as previously described [20,21,22]. The relative intensity of the indicated band was quantified using Image J software (1.50i; Java 1.6.0_24, National Institutes of Health, Bethesda, MD, USA), and the value was normalized to a corresponding loading control and expressed as the average value from three independent experiments.

### 2.8. Statistical Analysis

All data were derived from at least three independent treatment repetitions. The results are expressed as the mean ± SD. The data were analyzed by ANOVA followed by Tukey’s test using GraphPad Prism 6 software (San Diego, CA, USA), and statistical significance was set at *p* < 0.05.

## 3. Results

### 3.1. Comparison of Antiproliferative Effects of Each Fraction

In a previous study, we examined the effect of the MeOH extract of the rhizome of *D. tokoro* on the proliferation of HepG2 cells [20]. The cells were treated with 5, 10, and 20 μg/mL MeOH extract for 24 h, and cell proliferation was measured using the MTT assay. The cell viability after treatment with 5, 10, or 20 μg/mL MeOH extract was 71.8%, 50.1%, and 20.0%, respectively. Among the approximately 130 crude drugs that we screened, the MeOH extract of the rhizome of *D. tokoro* exhibited the strongest antiproliferative effect.

**Figure 1:**
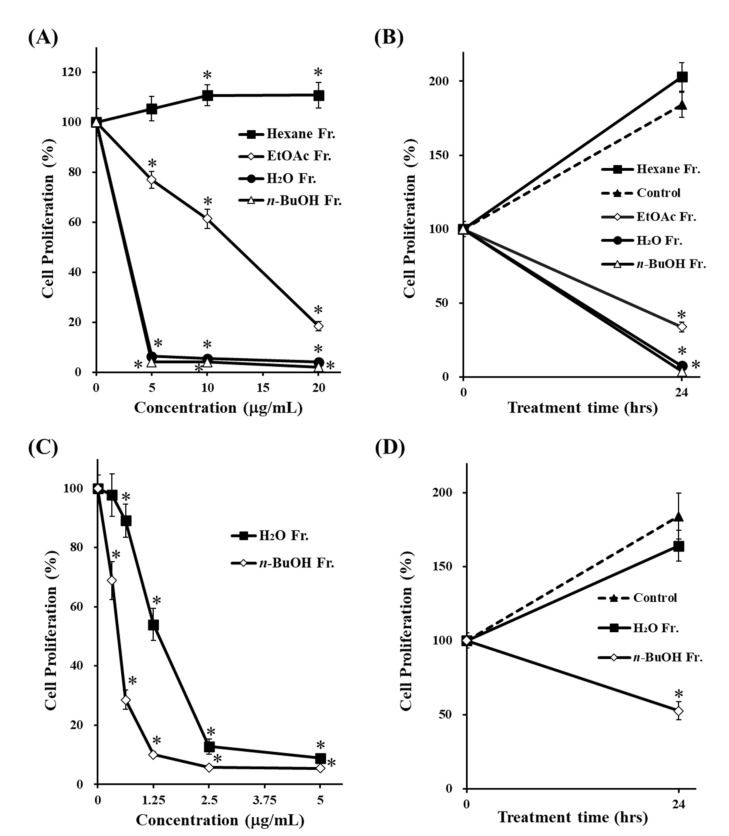
Effects of fractions prepared from MeOH extract of the rhizome of *D. tokoro* on cell proliferation. HepG2 cells were treated with various concentrations of each fraction (**A**) 5–20 μg/mL, (**C**) 0.313–5 μg/mL) for 24 h. Cells were treated with DMSO, *n*-BuOH or H_2_O fractions (**B**) 20 μg/mL, (**D**) 0.625 μg/mL) for 24 h and cell proliferation was determined using the MTT assay. The data are represented as the mean ± SD of three individual experiments. (**A**,**C**); * *p* < 0.05 compared with the non-treated cells (concentration 0 μg/mL). (**B**,**D**); * *p* < 0.05 compared with the non-treated cells (control) at 24-h incubation.

To identify the bioactive compounds responsible for the antiproliferative effect, the crude MeOH extract was suspended in water and successively partitioned using hexane, EtOAc, and *n*-BuOH. The cells were treated with 5, 10, or 20 μg/mL of each fraction for 24 h, followed by the MTT assay. The cell proliferation in Figure 1A,C was expressed as a percentage of control culture at 24-h incubation. In contrast, the cell proliferation in Figure 1B,D was expressed as a percentage of control culture at beginning of incubation (0 h), indicating that 24-h incubation increased the cell proliferation more than 150% in non-treated cells (control). As shown in Figure 1A,B, both *n*-BuOH and H_2_O fractions significantly suppressed cell proliferation compared to the hexane and EtOAc fractions. To further determine which fraction exerted the stronger effect, the cells were treated at concentrations much lower than 5 μg/mL of the *n*-BuOH and H_2_O fractions for 24 h (Figure 1C,D). The results showed that the *n*-BuOH fraction had a stronger antiproliferative effect than the H_2_O fraction.

### 3.2. Effect of the n-BuOH Fraction on Autophagosome Formation and Autophagy Flux

**Figure 2:**
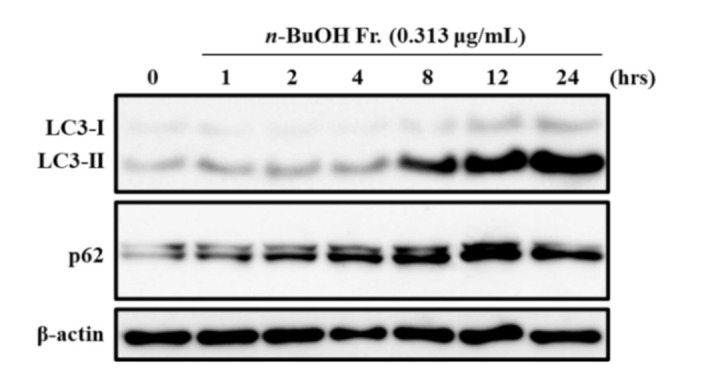
Effect of the *n*-BuOH fraction on LC3-II and p62 expression levels. HepG2 cells were treated with 0.313 μg/mL of the *n*-BuOH fraction at for the times indicated. The expression levels of LC3B, p62, and β-actin were determined by Western blotting. The data shown are representative of three independent treatments using the same parameters with similar results. (Original Western Blotting Figure see Supplementary Materials).

To confirm the effect of the *n*-BuOH fraction on autophagy, we measured the expression levels of both LC3-II and p62. The cells were treated with 0.313 μg/mL of the *n*-BuOH fraction for various time periods, and the levels of each protein were examined by Western blotting. As shown in Figure 2, treatment with the *n*-BuOH fraction clearly increased the levels of both LC3-II and p62. These results suggest that the *n*-BuOH fraction might inhibit autophagy by blocking autophagic flux, resulting in p62 and LC3-II accumulation.

### 3.3. Isolation, Acid Hydrolyses, and Structural Identification of ***1***–***5***

**Figure 3:**
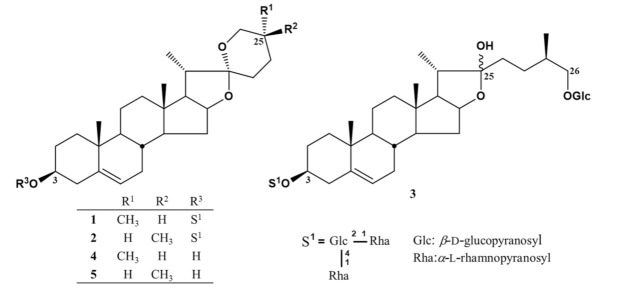
Chemical structures of **1**–**5**.

Since the *n*-BuOH fraction reduced cell proliferation most strongly and inhibited autophagy, we isolated bioactive compounds from this fraction. Further bioassay-guided separation using solvent partition, fractionation, and combined chromatography techniques resulted in the isolation of three compounds from the *n*-BuOH fraction. We isolated two spirostan-type steroidal saponins, dioscin (**1**) [26,27] and yamogenin 3-*O*-*α*-l-rhamnopyranosyl(1→4)-*O*-*α*-l-rhamnopyranosyl (1→2)-*β*-d-glucopyranoside (**2**) [26,28,29], and the frostane-type steroidal saponin protodioscin (**3**) [26,30]. The structures were identified by physicochemical data obtained using NMR and MS, together with a comparison with those in the literature. In addition, **1** and **2** were acid-hydrolyzed to obtain aglycone, diosgenin (**4**) [26,31], and yamogenin (**5**) [26,32]. The chemical structures of compounds **1**–**5** are shown in Figure 3. Among them, **1** and **3** are steroidal saponins that are well known as the main compounds in the rhizome of *D. tokoro* [24,25,33].

### 3.4. Effects of ***1***–***5*** on Cell Proliferation, and their Structure–activity Relationships

**Figure 4:**
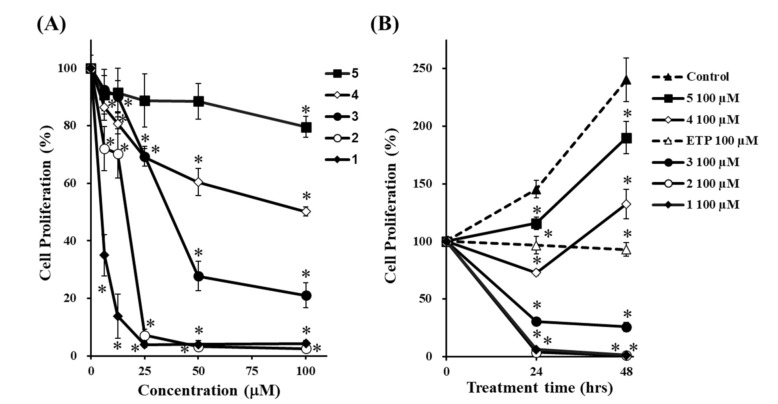
Effects of **1**–**5** on cell proliferation. HepG2 cells were treated with **1**–**5** at various concentrations for 24 h (**A**), and with **1**–**5** (100 µM) for the times indicated (**B**), and cell proliferation was determined using the MTT assay. ETP was used as a positive control for inducing apoptotic death of HepG2 cells. The data are presented as the mean ± S.D. of three individual experiments. (**A**); * *p* < 0.05 compared with the non-treated cells (concentration 0 μM). (**B**); * *p* < 0.05 compared with the non-treated cells (control) at 24- or 48-h incubation.

To investigate the effects of **1**–**5** on the proliferation of HepG2 cells, we treated them with 6.25, 12.5, 25, 50, and 100 μM of **1**–**5** for 24 h, followed by the MTT assay. Etoposide (ETP) was used as a positive control [34,35]. The cell proliferation in Figure 4A,B was expressed as a percentage of control culture at 24-h incubation and at beginning of incubation (0 h), respectively. As shown in Figure 4B, the cell proliferation of the non-treated cells (control) at 24 h and 48 h were approximately 150% and 250%, respectively. On the other hand, the cell proliferation of ETP at 24 h and 48 h were approximately 100%, suggesting that ETP strongly suppressed the cell proliferation. When we compared the antiproliferative effect of each compound, all compounds reduced cell proliferation and the potency was in the following order: **1** > **2** > **3** > **4** > **5** (Figure 4A,B).

### 3.5. Effects of ***1***–***5*** on Autophagosome Formation and Autophagy Flux

**Figure 5:**
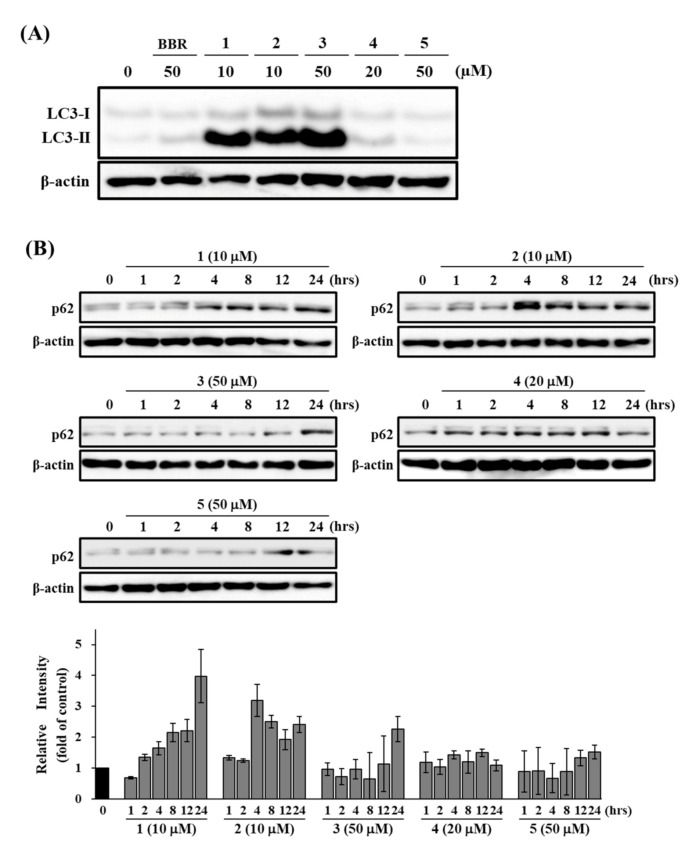
Effects of **1**–**5** on LC3-II and p62 expression levels. HepG2 cells were treated with the indicated concentrations of **1**–**5** for 24 h (**A**), or different durations (**B**). The expression levels of LC3B (**A**), p62 (**B**), and β-actin were determined by Western blotting. BBR (50 μM) was a positive control. The data shown are representative of three independent treatments using the same parameters with similar results. (Original Western Blotting Figure see Supplementary Materials).

To determine whether **1**–**5** inhibited autophagy, we investigated their effects on the levels of both LC3-II and p62 in HepG2 cells. The cells were treated with **1** and **2** at 10 μM, **3** and **5** at 50 μM, and **4** at 20 μM for various time periods. Berberine (BBR), which is known to incuce autophagy in HepG2, was used as a positive control for LC3-II [36]. As shown in Figure 5, treatment with **1**–**3** clearly increased the levels of both LC3-II and p62, although BBR slightly induced LC3-II after 24 h of incubation. The induction of LC3-II by **1**–**3** was higher than that by BBR. These results suggest that **1**–**3** deregulate the autophagic pathway by blocking autophagic flux, which results in p62 and LC3-II accumulation. In contrast, treatment with **4** and **5** did not show a clear increase in the levels of both LC3-II and p62. These results suggested that **4** and **5** did not affect autophagy.

### 3.6. Effects of **1**–**3** on Apoptosis-Related proteins

**Figure 6:**
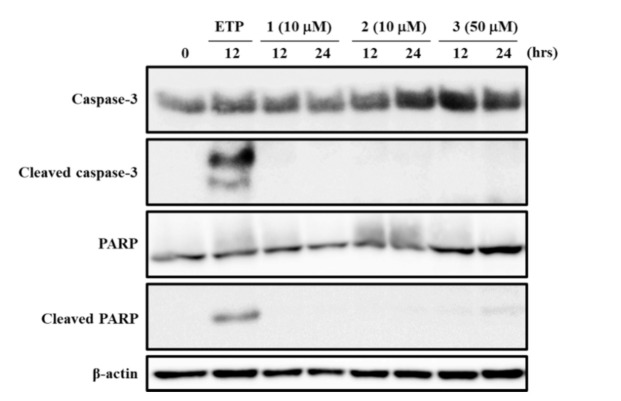
Effects of **1**–**3** on the activation of caspase-3 and PARP cleavage. HepG2 cells were treated with the indicated concentrations of **1**–**3** for 12 or 24 h. The expression levels of caspase-3, PARP, and β-actin were determined by Western blotting. ETP (100 μM, 12 h) was used as a positive control. The data shown are representative of three independent treatments using the same parameters with similar results. (Original Western Blotting Figure see Supplementary Materials).

Caspase-3 activation and PARP cleavage are characteristic hallmarks of apoptotic responses [23,34,37]. To determine whether the high antiproliferative effects of **1**–**3** are associated with apoptosis induction, we examined their effects on caspase-3 and PARP. ETP was used as a positive control to induce apoptotic cell death in HepG2 cells [34,35]. As shown in Figure 6, treatment with **1** and **2** at 10 μM, and **3** at 50 μM for 12 and 24 h did not induce the activation of caspase-3 and cleaved PARP. These results suggested that treatment with **1**–**3** did not induce caspase-3-mediated apoptosis in HepG2 cells.

## 4. Discussion

To develop future cancer therapy strategies, additional research is required to elucidate the role of autophagy in cancer. However, there are few reports on the isolation of compounds that modulate autophagy from natural products. In our previous study, 130 crude drugs were screened, and the MeOH extract of the rhizome of *D. tokoro* exhibited the strongest antiproliferative effect on HepG2 cells [20]. Furthermore, this extract significantly increased LC3-II expression and p62 accumulation. There is no report on the bioactive compounds in *D. tokoro* regulating autophagy; therefore, this study aimed to isolate active compounds from the rhizome that inhibit proliferation and autophagy.

Among the four fractions prepared from the MeOH extract of the rhizome of *D. tokoro*, the *n*-BuOH fraction exhibited the strongest antiproliferative effect compared to the other three fractions (Figure 1). In addition, the *n*-BuOH fraction increased the levels of both LC3-II and p62, leading to the inhibition of autophagy (Figure 2). The bioassay-guided fractionation of the *n*-BuOH fraction led to the isolation of three compounds (**1**–**3**). Furthermore, acid hydrolysis of **1** and **2** produced the aglycones **4** and **5**, respectively. Among **1**–**5**, **1** and **3** are well-known as the main compounds in the rhizome of *D. tokoro* [24,25]. Compounds **1**–**3** are steroidal saponins, and compounds **4** and **5** are produced by acid hydrolysis of **1** and **2** (Figure 3). According to the results of cell proliferation analysis in HepG2 cells, **1**–**5** exerted antiproliferative activity, and the potency was in the following order: **1** > **2** > **3** > **4** > **5** (Figure 4). The effects of **1** and **4** with a 25(*R*)-conformation were stronger than those of **2** and **5** with a 25(*S*)-conformation. These results indicate that the efficacy of the 25(*R*)-conformation was maintained with or without the sugar moiety. Next, the effects of **1**–**3** containing a sugar moiety were found to be stronger than those of **4** and **5**, which lack one. In addition, compounds **1** and **2**, which possess a spirostan-type aglycone moiety, showed stronger effects than compound **3**, which possesses a furostan-type aglycone moiety. From these results of structure–activity relationships, it was concluded that the 25(*R*)-conformation, structures containing a sugar moiety, and spirostan-type aglycone moiety might be important for antiproliferative effects. To confirm that **1**–**5** inhibited autophagy in HepG2 cells, autophagic flux was assessed by monitoring p62 levels upon treatment with **1**–**5** for different times. Treatment with **1**–**3** deregulated the autophagic pathway by blocking autophagic flux, which resulted in p62 accumulation (Figure 5). Similarly, the levels of LC3-II were increased upon treatment with **1**–**3**, but not after treatment with **4** and **5**. These results suggest the importance of structures containing a sugar moiety in modulating autophagy with LC3-II and p62 accumulation. Although compounds **1**–**3** exerted inhibitory activity on autophagy, these compounds did not activate caspase-3 and cleavage of PARP, which are characteristics of apoptosis induction (Figure 6). Taken together, we conclude that the antiproliferative activity of **1**–**3** might be due to the inhibition of autophagy, but not to the induction of caspase-3–mediated apoptosis. These findings provide new insights into the function of steroidal saponins from the rhizome of *D. tokoro* with respect to autophagic activity in hepatocellular carcinoma cells. In addition, our results indicate that these steroidal saponins might be used as autophagy inhibitors to analyze the mechanism of autophagy.

Chloroquine (CQ), hydroxychloroquine (HCQ), bafilomycin A1, ammonium chloride, and Lys05 are known as suppressors at the late-stage of autophagy [14,38,39,40]. HCQ, an analog of CQ, is used for clinical trials to develop anticancer drug [14,38,39,40]. The chemical structures of compounds **1**–**3** differ from existing those of drugs used as inhibitors of autophagy, which may lead to the development of new autophagy inhibitor. Further studies using several hepatoma cell lines or an animal model are needed to confirm our findings and develop new clinical therapies. Compound **1** was reported to induce apoptosis via the mitochondrial pathway in various cancer cell lines [24]. The treatment with **1** triggars the loss of mitochondrial membrane potential [41], downlegulation of Bcl-2 and Bcl-xL expressions [42], upregulation of Bax and Bak expressions [42], activation of caspase-9, caspase-7, and caspase-3 [43], and releases of cytochrome c into the cytosol [41,42,43]. It also induces DNA damage mediated by ROS [44,45]. Proteomic study inducated that **1** is involved in oxidative phosphorylation, and in Wnt, p53, and calcium signaling pathways [44]. Furethermore, it was reported that **1** enhances osteoblastic cell differentiation and proliferation by inhibiting the autophagy via the apoptosis stimulated protein of p53–2 (ASPP2)/NF-κβ pathway [46]. Additional research will be necessary to evaluate the mechanism of action by **1**–**3** involved in the inhibiton of cell growth and autophagy in hepatocellular carcinoma cells autophagy.

## 5. Conclusions

We found that *n*-BuOH and H_2_O fractions of the rhizome of *D. tokoro* exerted strong antiproliferative activity and inhibited autophagy in HepG2 cells. Phytochemical investigations of the *n*-BuOH fraction resulted in the isolation of steroidal saponins **1**–**3**. In addition, acid hydrolysis of **1** and **2** produced the aglycones **4** and **5**, respectively. Structure-activity relationship analysis indicated that the 25(*R*)-conformation, structures containing a sugar moiety, and a spirostan-type aglycone moiety are important for antiproliferative activity. Analysis of autophagy-related proteins in HepG2 cells demonstrated that **1**–**3** inhibited autophagy, leading to p62 accumulation. In contrast, **1**–**3** did not affect caspase-3 activation and PARP cleavage, suggesting that the antiproliferative activity of **1**–**3** can occur independently of caspase-3–mediated apoptosis. Taking these findings together, we conclude that **1**–**3**, active compounds in the rhizome of *D. tokoro*, suppress cell proliferation and inhibit autophagy.

## Data Availability

Data in this study will be provided upon reasonable request to the corresponding author.

## Supplementary Materials

The following are available online at https://www.mdpi.com/article/10.3390/life11080749/s1.
