# Supplementary figures and images for: Steroidal Saponins Isolated from the Rhizome of Dioscorea tokoro Inhibit Cell Growth and Autophagy in Hepatocellular Carcinoma Cells
Source: Life (Basel). 2021 Jul 26;11(8):749. doi: 10.3390/life11080749 (PMC8400091; doi:10.3390/life11080749)

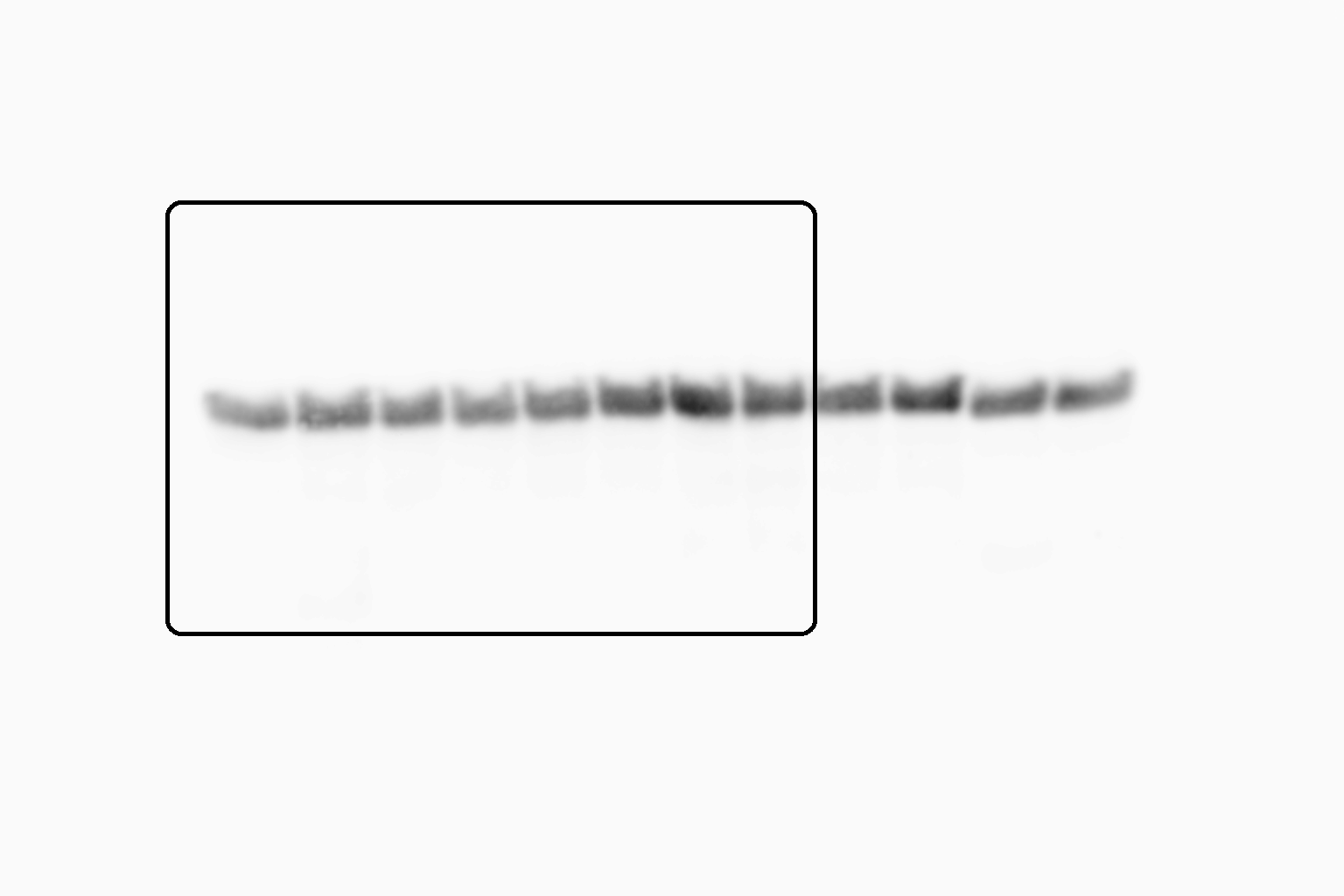

Supplement: Supplementary file 1 [file life-11-00749-s001.zip › 16) Fig.6 Caspase-3.tif]

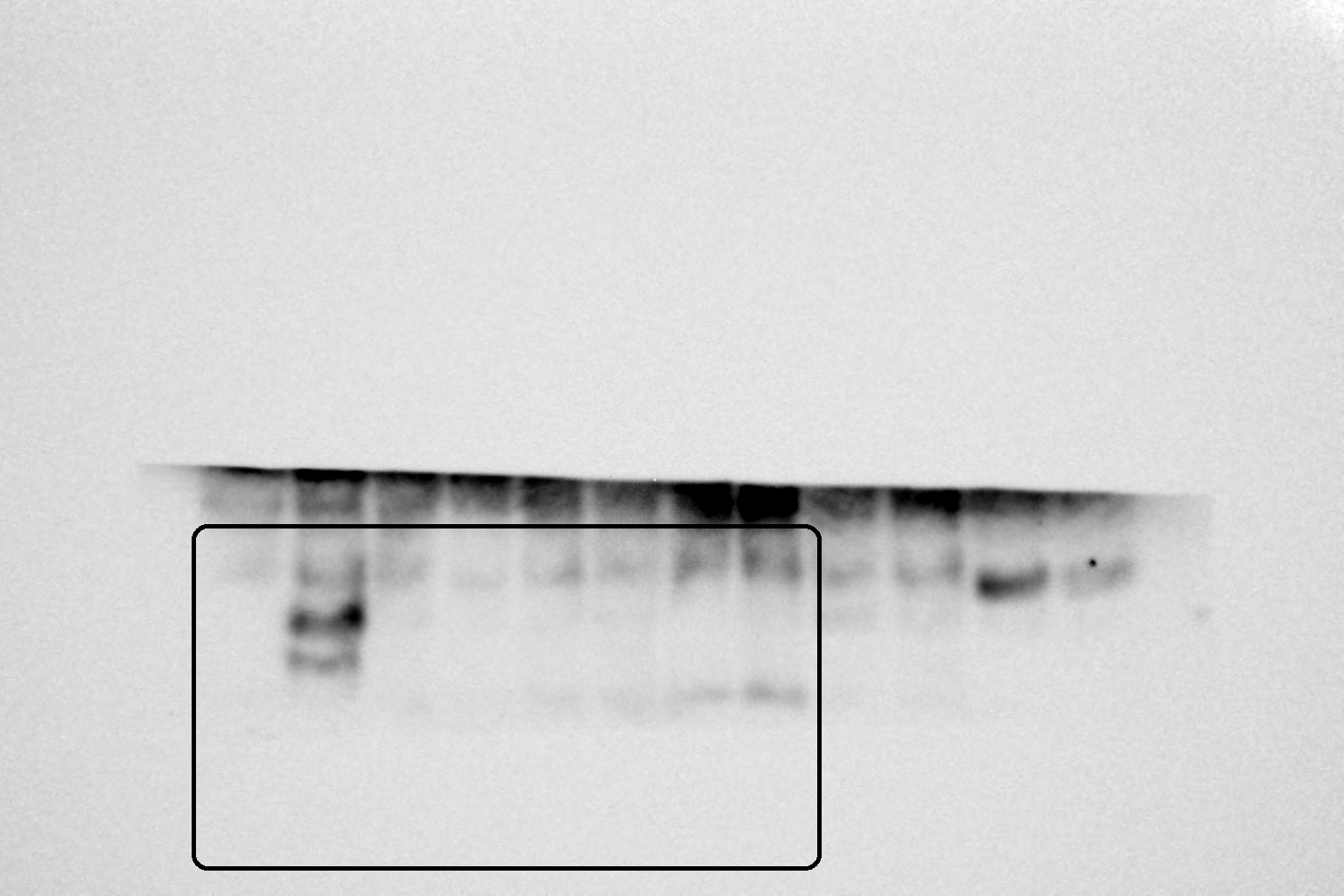

Supplement: Supplementary file 1 [file life-11-00749-s001.zip › 17) Fig.6 Cleaved caspase-3.tif]

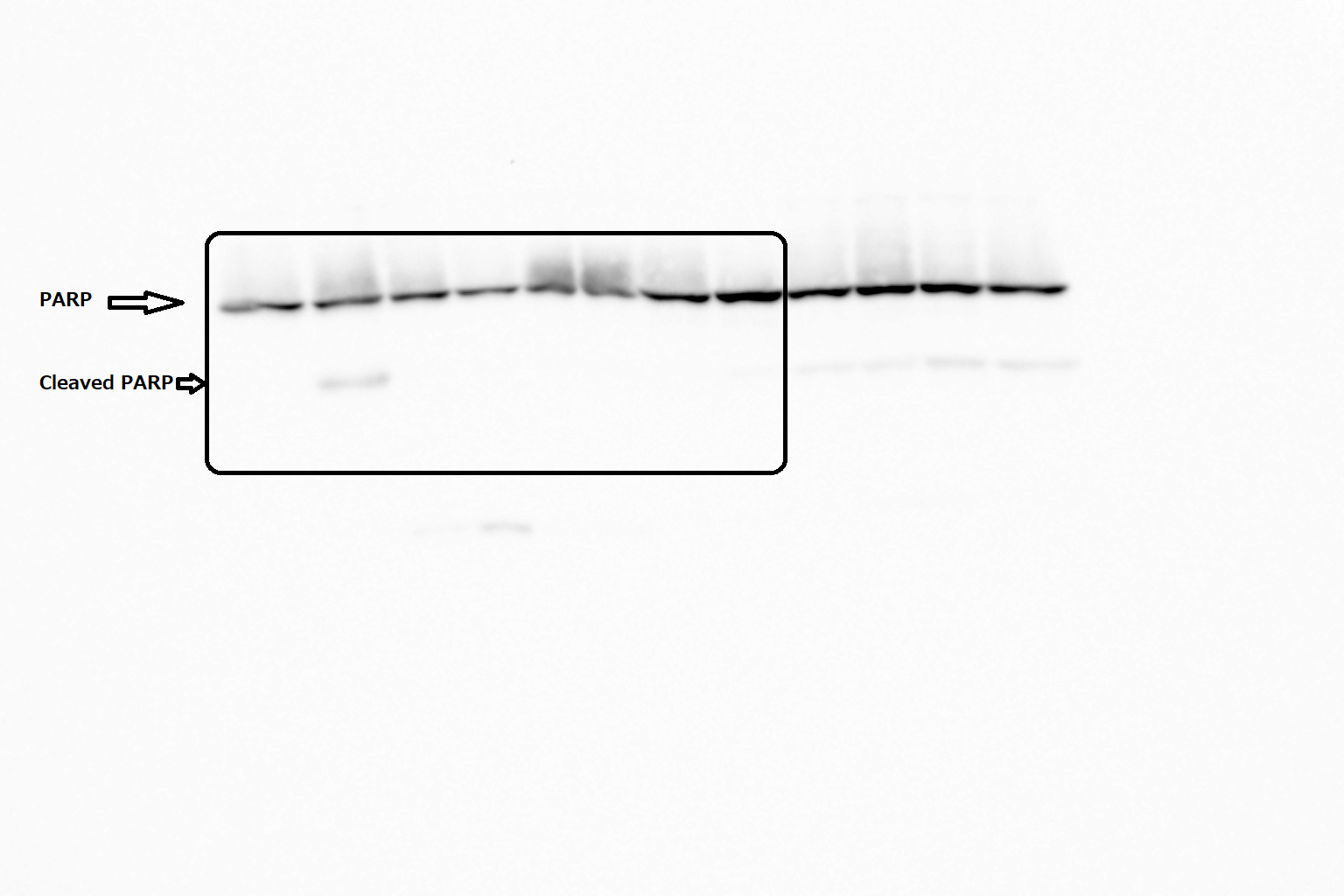

Supplement: Supplementary file 1 [file life-11-00749-s001.zip › 18and19) Fig.6 PARP and Cleaved PARP.tif]
